# Multi-Vehicle Tracking via Real-Time Detection Probes and a Markov Decision Process Policy

**DOI:** 10.3390/s19061309

**Published:** 2019-03-15

**Authors:** Yi Zou, Weiwei Zhang, Wendi Weng, Zhengyun Meng

**Affiliations:** College of Mechanical and Automotive Engineering, Shanghai University of Engineering Science, Shanghai 201620, China; zoezou9@163.com (Y.Z.); wangxinchensues@163.com (W.W.); mzysues@163.com (Z.M.)

**Keywords:** tracking-by-detection, multi-vehicle tracking, Siamese network, data association, Markov decision process

## Abstract

Online multi-object tracking (MOT) has broad applications in time-critical video analysis scenarios such as advanced driver-assistance systems (ADASs) and autonomous driving. In this paper, the proposed system aims at tracking multiple vehicles in the front view of an onboard monocular camera. The vehicle detection probes are customized to generate high precision detection, which plays a basic role in the following tracking-by-detection method. A novel Siamese network with a spatial pyramid pooling (SPP) layer is applied to calculate pairwise appearance similarity. The motion model captured from the refined bounding box provides the relative movements and aspects. The online-learned policy treats each tracking period as a Markov decision process (MDP) to maintain long-term, robust tracking. The proposed method is validated in a moving vehicle with an onboard NVIDIA Jetson TX2 and returns real-time speeds. Compared with other methods on KITTI and self-collected datasets, our method achieves significant performance in terms of the “Mostly-tracked”, “Fragmentation”, and “ID switch” variables.

## 1. Introduction

Advanced driver-assistance systems (ADASs) and autonomous driving have consistently been a popular research area. An intelligent vehicle is expected to interact with other vehicles as well as other traffic participants, in which case relative movement tendencies of a multi-vehicle environment is of great concern. An accurate multi-vehicle tracker is necessary for several tasks such as location, navigation, and traffic behavior analysis.

In the research area of single-object tracking (SOT), most state-of-the-art methods tend to learn a discriminative classifier on labeled sample patches within a neighborhood area [1,2,3]. Especially, when deep neural networks (DNNs) show powerful effectiveness in feature selection, the performance of tracking significantly improves [4,5,6]. Multi-object tracking (MOT) comes from SOT, and it has wide applications in visual surveillance, traffic monitoring [7,8,9], sports analysis, ADAS, and autonomous driving. The goal of MOT is to estimate the locations of multiple objects in real-time while maintaining each identity consistently and yielding individual trajectories [10,11,12,13]. However, multi-object tracking faces special challenges that can be even more serious with moving camera platforms. Firstly, multiple targets may share a similar appearance in complex scenarios, and appearance may change dramatically at any time. Secondly, observable motion cues are more complicated since new emerging targets and tracked targets always overlap with each other. When it comes to onboard moving camera platforms, these conditions deteriorate, and tracking models need to put more computational overhead on real-time performance. All the above factors contribute to tracking drift and even failure.

Multi-object tracking benefits significantly from advances in object detection in recent years. Tracking-by-detection frameworks [3,11,12,14,15,16] have achieved extremely reliable test results and have shown great potential in handling object appearance variations and model drifts. Distinguished from the detection-free tracking method that needs to calibrate targets manually first, the tracking-by-detection approach is more feasible in handling new targets at each time step in a dynamic environment. This kind of approach detects objects in each frame and then matches them in the following frames to form complete trajectories. The batch tracking system [12,14,17] utilizes a set of detection results collected by temporal sliding windows of whole frames to generate global trajectories. Although such offline tracking methods perform well in obtaining an optimal, theoretical global solution in partial time snippets, they are not applicable in handling dramatic model changes in online, long-term tracking. Specifically, the real-time tracking application requires online methods [16,18,19,20] to handle up-to-time observations and sequentially extend existing trajectories with current detections based on frame-by-frame associations.

Date association and matching play a vital role in MOT identity assignment. The Hungarian method [21] is applied to achieve matching of bipartite graphs by finding the minimum point solution of the assignment matrices. The feature of appearance (e.g., color histogram, histogram of oriented gradients (HOG) feature, shapes feature, texture, and optical flow) is usually extracted as a part of a measurement. The rigid characteristics of vehicles benefits this under positive conditions for generating discriminative appearance models in data association. Inspired by multiple neural network architectures [22], the two-channel network is used to learn a richer hierarchical feature of patches and output pairwise similarity. Moreover, combined with spatial pyramid pooling (SPP) layers [23], the network reduces the size limitation, and thus becomes more reasonable in practice.

On the other hand, there are inaccurate detections of occluded and novel objects, so the process of learning to track is a trend that can deal with these ambiguities in data association [15,16,18,19,24,25,26]. In this study of tracking with a moving camera, scenarios are more complex and unpredictable. ID switch is one of the most common problems in long-term tracking, where the previous methods are less reliable to handle. In order to improve long-term tracking robustness, a Markov decision processes (MDP) is introduced to manage the state of each object and alleviate track drift. Furthermore, reinforcement learning is applied to learn data association policies, which could effectively cope with the appearance/disappearance of each vehicle by state transition.

In this paper, an integrated framework is proposed to track frontal vehicles with an onboard monocular camera, which can assist intelligent vehicles with substantial benefits in high-performance and safe distance maintenance. The main contributions of this paper are threefold:

- An offline-trained vehicle detector is customized to generate robust and fine detections by an onboard monocular camera. Data augmentation benefits the detector to meet various traffic conditions in moving scenes.
- A well-designed association strategy adopts multi-dimensional information to score pairwise similarity. A Siamese convolution network is designed to score pairwise similarity, wherein a dual-resolution in two specific channels could efficiently improve the performance of image matching. Any size of the input patches can still maintain the fixed output dimensionality through the SPP layer. A tracking-by-detection framework is applied to accomplish linear assignments by linking new detections with initial tracks.
- The tracking process is formulated as the Markov decision process. Four states are designed to manage the lifetime of each vehicle, which is more adaptable to the changeable traffic scenes. With reinforcement learning, an updated policy is applied to reduce false positives and improve tracking accuracy.

The rest of this paper is organized as follows. Related work is discussed in Section 2. Section 3 describes the specific methods from three parts in details. Experimental results are analyzed in Section 4, and Section 5 concludes the paper.

## 2. Related Work

Recently, the tracking-by-detection framework has become the leading paradigm in MOT because of its remarkable processes in object detection. These approaches formulate MOT as a data association problem, in which the main task is linking individual detections to build longer tracklets. Sadeghian et al. [15] followed this paradigm, whereby temporal detections were encoded across appearance, motion, and interactions for tracking multiple targets. In [26], a continuous confidence of detectors was proposed, and then target-specific classifiers were learned to select high-confidence detections and were associated to targets for robustly tracking multiple people in complex scenes. Coifman et al. [7] proposed a video image processing system to realize effective traffic surveillance. They took corner points of vehicles as the relevant feature, which made the system less sensitive to partial occlusions. Bae and Yoon [20] formulate an MOT problem based on tracklet confidence, in which fragmented tracklets were linked up with others, relying on online-provided detections. Sanchez-Matilla et al. [25] associated strong and weak detection responses for tracking, which denoted that high confidence detections could initialize targets while weak confidence detections only supported the propagation of labels. In this work, the tracking task of each vehicle is initialized frame-by-frame according to the latest detections.

The core of multi-object tracking is based on data association, which is to identify correspondence between trajectories and new detections. The key in corresponding is how to compute a matching score that models multiple cues from the past, such as object interactions, appearances, and motions. A tracking method based on the template matching was reported in [8], which can dynamically switch modules to handle various conditions in real sequences. Yoon et al. [16] utilized a structural model to realize the best assignment by minimizing total cost, in which an event aggregation approach was developed to integrate structural constraints in assignment cost. However, it showed limited camera motion performance because a single metric model was used. The association cost in [25] relied only on the position and size, so nearby targets were hard to discriminate. Besides motion information, Wojke et al. [27] integrated an appearance model and a deep association metric, which was trained on a large-scale person re-identification dataset to improve the performance of real-time tracking [28]. In [20], both tracklet confidence and learned-appearance models were designed to support a reliable association for multi-object tracking problems. In such methods above, the Hungarian algorithm [21] helps to solve the bipartite matching problem of possible tracker-detection anchors.

Bromley et al. [29] proposed a two-stream Siamese architecture for signature verification. Similarly, this architecture was introduced for face verification in [30], where two identical convolutional networks were trained to realize similarity metric learning. Inspired by successful progress in the convolutional neural network, deep neural networks are employed in Siamese invariance networks to learn the generic matching function for single object tracking. Tao et al. [31] focused on the learning strategy of matching functions, but they had a large gap in handling specific MOT problems, e.g., occlusion or model update. In this multi-vehicle tracking task, an improved Siamese network with a dual-resolution stream is used to generate similarity between pairs of candidates for data association. Specifically, an SPP layer [23] is embedded to release size constraints by fixed dimensional characteristics. Consequently, the network becomes more variable in managing arbitrary patches in practical tracking scenarios.

Recently, the MDP [32] has been widely used in computer vision to learn policy parameters. Karayev et al. [33] found a dynamic policy of optimizing feature selection and classification strategies by formulating the problem as an (MDP). Kitani et al. [34] incorporated uncertainty and noise observations into the hidden variable MDP (hMDP) model to realize activity understanding and forecasting in computer vision. In [35], in order to balance the cost and accuracy in the study of human–machine collaboration in object annotation, the MDP was used to automatically quantify the best tradeoff. Inspired by previous research, the proposed state transition framework is designed to manage each single object tracker as a separate agent in MDP. Each action is responsible for a specific situation, such as in false alarms and missed detection in cluttered traffic scenes. The potential for ambiguous tracking can be alleviated by correcting detection errors and recovering observations from an occluded period.

## 3. Methods

**Figure 1:**
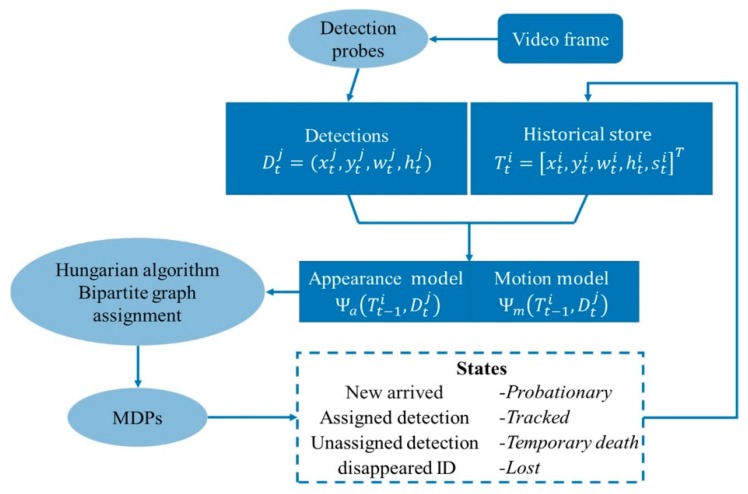
The overview of the proposed multiple vehicle tracking system. Discriminative appearance similarity and motion model are implemented to perform pairwise associations and Markov decision processes (MDPs) to define the real-time state.

The proposed tracking scheme consisted of detecting targets and matching their identities frame by frame, which led to a set of target trajectories over time. The tracking-by-detection method was used to address this problem. Figure 1 shows the overview of the proposed multiple-vehicle tracking framework. The detection probes produced simultaneous current results, and the tracker guaranteed long-term tracking. New detections were linked to the activated tracks at each time step by solving the linear assignment problem. The motion and appearance model were integrated to create a pairwise matching score matrix, where traditional methods and deep learning were both involved. The initialized targets $T_{t}^{i}$ and the new detections $D_{t}^{j}$ were gathered in a bipartite graph, and the Hungarian algorithm was used to find the optimal assignments that maximized the total matching score. Finally, to realize stable tracking, each object was initialized with its own MDP that could manage lifetime based on real-time state transition. Moreover, it relied on online reinforcement learning to learn a policy for data association between training tracks and ground truth.

### 3.1. Vehicle Detection Probes

Based on the tracking-by-detection framework, the robustness of the real-time tracking system takes advantage of high-precision detection results. The single shot detector YOLOv3 runs significantly faster than other detection methods, which makes it more suitable to be applied in real-time tasks. The proposed vehicle traction probes were trained based on YOLOv3 in rich datasets to improve the precision of vehicle detection.

The vehicle images formed the KITTI Vision Benchmark [33] and a self-collected dataset that were both integrated to increase the diversity of training samples, which involved multi-scale vehicles in different scenes containing occlusions and truncations. Furthermore, facing various appearances of vehicles in dynamic traffic scenes, data augmentation was adopted to improve generalization. Specifically, the brightness, contrast, and saturation of the images were changed to adapt to various light conditions. The straighten angle was rotated to deal with different tracking views. The training dataset contained a total of 18,952 images with 480 × 640 pixels, which contained various appearances of vehicles in different light conditions. Since the batch size was set to 50, one epoch needed to iterate 18,952/50 = 379 times. The training epochs were set to 60, and thus the number of iterations was 160 × 379 = 60,640. Different vehicle types, such as MPVs, SUVs, sedans, hatchbacks, vans, minibuses, pickups, and other types were trained to annotate as “vehicle”. Furthermore, an intersection over union threshold of 0.7 was adopted for evaluation. The precision of the bounding box was highly demanded while the position feature sets were used for calculating matching measurements. In this work, an iterative refinement framework [36,37] was conducted to improve localization accuracy by tight object-bounding boxes.

By comparing tracking performances by switching the detector component, the evaluation result could verify the effectiveness of the proposed detection probes, and it could demonstrate that detection quality plays a significant role in the tracking-by-detection framework for MOT.

### 3.2. Diversity Feature Extraction

The goal of data association is to identify the correspondence between pre-existing tracks and new detections. A set of linear corresponding constraints between an initialized trajectory $\;T_{t}^{i}$ and a current detection $D_{t}^{j}$ is defined to discriminate how well a pair of candidate patches match. Motion and appearance models are integrated into this problem formulation by addressing appropriate metrics.

#### 3.2.1. Motion and Size Models

Small changes in object positions are the critical components of data associations in traffic scenes. The motion model used the Mahalanobis distance to measure relative movements, which defines the distance between the initialized target $\;T_{t-1}^{i}$ and the current detection $D_{t}^{j}$. The bounding coordinates of initial and detected scenes are represented as: $T_{t-1}^{i}={\left(x_{t-1}^{i},y_{t-1}^{i},w_{t-1}^{i},h_{t-1}^{i}\right)}^{T}$, $D_{t}^{j}={\left(x_{t}^{j},y_{t}^{j},w_{t}^{j},h_{t}^{j}\right)}^{T}$,

(1) $$\begin{array}{c} d_{\left(T_{t-1}^{i},D_{t}^{j}\right)}=\sqrt{{\left(D-T\right)}^{Τ}\Sigma^{-1}\left(D-T\right)} \end{array}$$

(2) $$\begin{array}{c} \Sigma_{\;T_{t-1}^{i}D_{t}^{j}}=\left[\begin{array}{cc} E\left[\left(T_{t-1}^{i}-E\left[T_{t-1}^{i}\right]\right)\left(T_{t-1}^{i}-E\left[T_{t-1}^{i}\right]\right)\right] & E\left[\left(T_{t-1}^{i}-E\left[T_{t-1}^{i}\right]\right)\left(D_{t}^{j}-E\left[D_{t}^{j}\right]\right)\right]\\ E\left[\left(D_{t}^{j}-E\left[D_{t}^{j}\right]\right)\left(T_{t-1}^{i}-E\left[T_{t-1}^{i}\right]\right)\right] & E\left[\left(D_{t}^{j}-E\left[D_{t}^{j}\right]\right)\left(D_{t}^{j}-E\left[D_{t}^{j}\right]\right)\right] \end{array}\right] \end{array}$$

where j is the number of current detections in frame t, and $\left(x_{t}^{j},y_{t}^{j}\right)$ denotes the upper-left corner of the detection bounding box in the image. The width $w_{t}^{j}$ and the height $h_{t}^{j}$ correspond to the size of the bounding box. As the vehicle is rigid, the area scale and the aspect ratio of the bounding box are also considered. The area scale α and the aspect ratio r of the detection are computed by wh and $\frac{w}{h}$, respectively. Σ represents the covariance matrix in the Mahalanobis distance, where the operator E denotes the expected value of its argument.

Given a pairwise object patch, the similarity score of motion is obtained as follows:

(3) $$\begin{array}{c} \Psi_{m}\left(T_{t-1}^{i},D_{t}^{j}\right)=\frac{1}{d_{\left(T_{t-1}^{i},D_{t}^{j}\right)}+{\left(r_{j}-r_{i}\right)}^{2}+{\left(\alpha_{j}-\alpha_{i}\right)}^{2}} \end{array}$$

#### 3.2.2. Central-Surround Two-Channel Spatial Pyramid Pooling (SPP) Network

**Figure 2:**
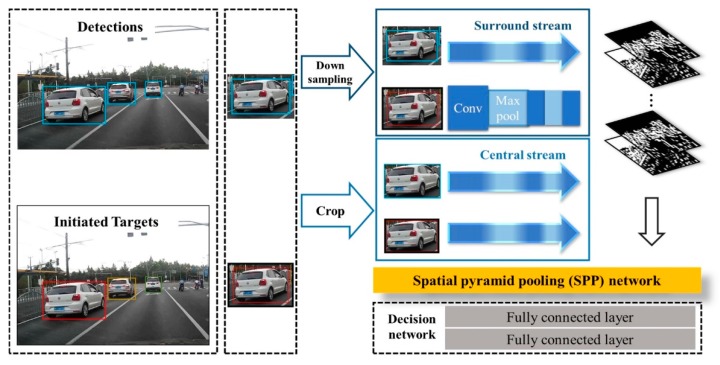
Central-surround two-channel spatial pyramid pooling network (CSTCSPP). This network uses the Siamese-type architecture to extract shallow features with different resolutions and then calculates pairwise similarity. A spatial pyramid pooling layer embedded before the top decision network allows patches to be free of size limitations. All convolution layers are followed by Rectified Linear Units (ReLU), which could increase the nonlinear relation between each layer of the neural network.

In the data association process, the similarity of appearance is definitely a crucial cue in matching score computations. In this section, a Siamese network was designed to compare corresponding targets and to output their pairwise similarities for discriminative appearance models. The framework is presented in Figure 2, and Table 1 details the architecture of each convolutional layer.

The so-called two-stream network was constructed of a central stream and a surrounding stream. It enabled this process in a spatial domain, in which two different resolutions were applied. The inputs of the network were pairs of image patches from the initial identity store and scaled current detection results. Besides the area caught by the tight bounding box, the surrounding environment also mattered to combat any similar appearances. The architecture of the network was inspired by VGG-M, which contained two branches with exactly the same set of weights. Different branches played unique roles in feature extraction functions.

To calculate similarity in the two-channel network, the patches of each target were cropped to (x−0.15w, y+0.15h, 1.3w, 1.3h) by experimental experience. Surrounding context features could enhance comparability, and large expansion may not only increase computation but also decrease accuracy. These patches go through down-sampling or cropping processes, and they are then transferred into the surrounding and central steams, respectively. Down-sampled patches in the surrounding low-resolution stream match the surrounding context features when the targets have a similar appearance. High-resolution patches in the central stream supplied more details about vehicle features. Two streams were designed to extract discriminative features, where the pixels of the vehicle and the periphery were all taken into consideration.

The prevalent convolutional neural networks (CNNs) require a fixed input image size due to the definition of the fully-connected layers, which limits both the aspect ratio and the scale of the inputs. In practical tracking scenarios, the detection patches are caught with arbitrary sizes under different distances and angles. With the help of a spatial pyramid pooling (SPP) layer, the network could aggregate features through spatial pooling and then generate a fixed-length representation. The top decision network consisted of two linear, fully connected layers with 512 hidden units. They were separated by the ReLU activation layer, which could increase the non-linearities inside the network and make the decision function more discriminative.

The parameters of the network were trained offline, based on self-collected datasets. In order to improve the efficiency in retrieving patch pairs and storing all the input images in Graphics Processing Unit (GPU) memory, data augmentation and preprocessing were adopted to train the model. The training data were augmented by flipping both patches horizontally and vertically and operating multi-degree rotation to reduce overfitting problems.

The learning function is calculated based on the $L_{2}$-norm regularization and hinge loss:

(4) $$\begin{array}{c} J\left(\omega \right)=\underset{\omega }{\min}\frac{\lambda }{2}{\left\|\omega \right\|}_{2}+\overset{N}{\underset{i=1}{\sum }}\max\left(0,1-y_{i}\mu_{i}\right) \end{array}$$

where ω is the weights of the neural network, $y_{i}\in \left\{-1,1\right\}$ is the corresponding label of the patch pairs with −1 and 1 denoting a non-matching and a matching pair, respectively. And $\mu_{i}\in \left(-1,1\right)$ represents the network output for the i–th training sample. Asynchronous stochastic gradient descent (ASGD) with a constant learning rate 1.0, momentum of 0.9, and weight decay of λ = 0.0005 was used to train the models. Weights were initialized randomly and all models were trained from scratch.

#### 3.2.3. Feature Representation

Constitute a tracklets historical store $T_{t}=\left\{T_{t}^{1},T_{t}^{2},\ldots ,T_{t}^{i}\right\}$, $T_{t}^{i}={\left[x_{t}^{i},y_{t}^{i},w_{t}^{i},h_{t}^{i},s_{t}^{i}\right]}^{T}$.

Where i is the number of initialized targets in the last frame t. Specifically, $T_{t}^{i}$ corresponded to the historical store of tracked targets in the previous frame, which contained multi-dimensional information about the location ${\left[x_{t}^{i},y_{t}^{i}\right]}^{T}$, the shape of bounding box ${\left[w_{t}^{i},h_{t}^{i}\right]}^{T}$, and the latest state ${\left[s_{t}^{i}\right]}^{T}$ in frame t. Generally, the store was preferable in this application, where facing dynamic situations involved false alarms and missed detections.

The similarity of motion

(5) $$\begin{array}{c} \Psi_{m}\left(T_{t-1}^{i},D_{t}^{j}\right)=\frac{1}{\eta d_{\left(T_{t-1}^{i},D_{t}^{j}\right)}+\delta {\left(r_{j}-r_{i}\right)}^{2}+\rho {\left(\alpha_{j}-\alpha_{i}\right)}^{2}}\; \end{array}$$

where η,δ,ρ are the weighing parameter to balance the value of distance, aspect ratio, and area scale, respectively. All parameters were found experimentally and remained unchanged for all datasets.

The similarity of appearance

(6) $$\begin{array}{c} \Psi_{a}\left(T_{t-1}^{i},D_{t}^{j}\right)\in \left(-1,1\right) \end{array}$$

The goal of data association is to find the set of trajectories $T_{t-1}$ that best explains the detections $D_{t}^{j}$. This means we needed to find the best linear assignment to get bipartite graph maximum matching scores. The matching score defined how probable a match was for pairwise objects between the tracked target and the current detection.

(7) $$\begin{array}{c} {Μ}_{\left(T_{t-1}^{i},D_{t}^{j}\right)}=max\left[\lambda \Psi_{m}\left(T_{t-1}^{i},D_{t}^{j}\right)+\left(1-\lambda \right)\left(\Psi_{a}\left(T_{t-1}^{i},D_{t}^{j}\right)+1\right)\right] \end{array}$$

Matching matrix

Consider a scenario where there are m preexisting tracks and n new detections at frame t. A matrix $M_{t}\in {\mathbb{R}}^{m\times n}$, which is ${Μ}_{\left(T_{t-1}^{i},D_{t}^{j}\right)}\in M$, represents the matching score of assigning detection j to track i at time t. The Hungarian algorithm was introduced to find the global optimal assignment matrix so that the total matching score was maximized.

### 3.3. Markov Decision Processes (MDPs)

This part focuses specifically on how to maintain robust multi-vehicle tracking, which is a tough challenge in MOT. Four states were utilized to handle false alarms and missed detections occurring in crowded scenes so that the tracker could re-identify the target with the same ID from any short-term occlusion.

#### 3.3.1. Overview of the MDPs

Due to multiple vehicles moving with varying speeds, inter-object occlusion and truncation often occurs in onboard, multi-object tracking tasks. Distinguished from SOT, multiple-object tracking depends on detection that often suffers from track drift when the appearance dramatically changes as a result of frequent inter-object occlusions.

**Figure 3:**
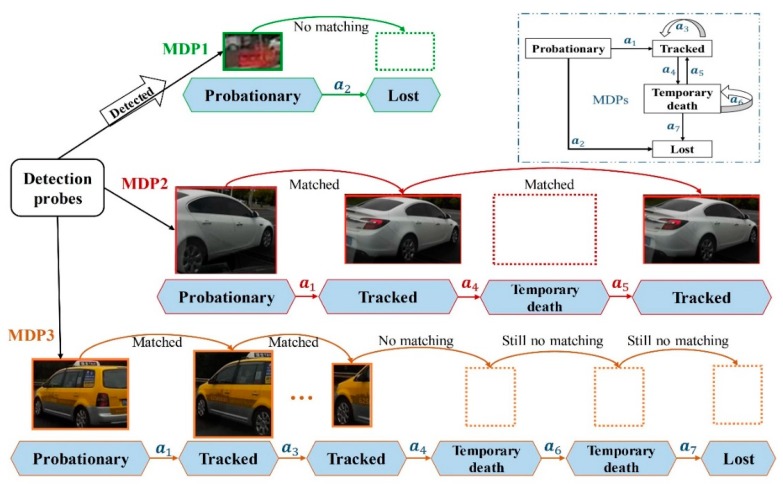
Online multi-vehicle tracking problem formulated as decision-making in MDP. The upper-right framework represents the transition map of four categorized states at each time step. Each target is initialized with a unique MDP to manage their lifetimes, depicted in different colors.

A Markov decision process (MDP) is the Markov reward process with a decision. In this framework, the lifetime of each target is modeled with an MDP that consists of four components (S,A,T(·),R(·)). s∈S encodes the status of the target in a particular time, which is determined by its previous action. Action a∈A can be performed to transfer the state in each frame. T represents the transition function, which can be described as T:S×A→S, and it describes the effect of each action in each state. R:S×A→ℝ defines the immediate reward received after executing action a to state s. Each target had its own corresponding MDP to handle the lifetime, and the process of state transition is detailed in Figure 3. Reinforcement learning provided a framework that was concerned with how the agent took action within a given state so as to maximize the cumulative reward.

The state space in the target MDP was parted into four subspaces, where each state encoded the global information of the target depending on feature representation, such as location, size, and appearance. Firstly, each object caught by the detector was activated to enter the “probationary” state. Vehicles in this state could transition to the “tracked” state only if they matched in the consecutive frames. Otherwise, the false alarm triggered entry to the “lost” state and removed the historical data. A tracked target could stay “tracked”, or transition into “temporary death” if the vehicle was lost due to occlusion by other vehicles, acceleration, or being out of view. Likewise, vehicles in the “temporary death” state had the chance to get back to “tracked” if it could complete successful matching, otherwise it transitioned to the “lost” state forever. Seven possible transitions were designed between the states of a target, which corresponded to seven actions in MDP.

#### 3.3.2. Policy in the Probationary State

Each detection that was unclaimed by any track underwent a probationary period where the target could be consistently detected to accumulate enough evidence. This period made up for the defect of false alarm and avoided an unnecessary increase of ID.

To handle targets in the probationary state, the MDP needed to decide whether it should switch to the “tracked” state or transfer into the “lost” state. If the tracked vehicles were not able to successfully associate any detection responses $D_{t}^{i}$ in the next consecutive frame, the MDP recognized the failure of tracking initialization, and transitioned the object to the “lost” state. In the meantime, redundant data was deleted for efficiency. Otherwise, the target finished the preprocessing step of tracking and was transferred to a “tracked” state.

This is equivalent to learning the reward function in the probationary period state:

(8) $$\begin{array}{c} R_{p}\left(s,a\right)=\{\begin{array}{c} y\left(a\right),\;if\;{Μ}_{\left(T_{t}^{i},D_{t}^{j}\right)}\geq m_{0}\;\\ -y\left(a\right),\;otherwise\; \end{array}, \end{array}$$

where y(a) = +1 if action a = $a_{1}$, and y(a) = −1 if a = $a_{2}$.

#### 3.3.3. Policy in the Tracked State

To handle targets in the tracked state, the MDP needed to decide whether to keep tracking or to transfer it to temporary death. If the activated trajectory could associate with the corresponding detection pair, the MDP recognized this target as still under tracking, otherwise transferred it to the “temporary death” state.

The reward function in the tracked state is defined as followed:

(9) $$\begin{array}{c} R_{tracked}\left(s,a\right)=\{\begin{array}{c} y\left(a\right),\;if\;{Μ}_{\left(T_{t}^{i},D_{t}^{j}\right)}\geq m_{0}\;\\ -y\left(a\right),\;otherwise\; \end{array}, \end{array}$$

where y(a) = +1 if action a = $a_{3}$, and y(a) = −1 if a = $a_{4}$.

#### 3.3.4. Policy in the Temporary Death State

In data association progress, unassociated tracks transitioned to the temporary death period. In addition, their coded feature and current state were historically stored just in case it was re-tracked (the red line in Figure 3). Trajectory terminated if they continued to fail to match with each input of detections, which meant this vehicle accelerated to speed away or was left behind (the yellow line in Figure 3). The linear function $ℒ\left(T_{t}^{i},D_{t}^{j}\right)=W^{T}\tau \left(T_{t}^{i},D_{t}^{j}\right)+b$ was used to make the decision rule. $\tau \left(T_{t}^{i},D_{t}^{j}\right)$ is the feature vector which represented the similarity between the initialized target and detection. Moreover, the coding message of the vehicle was deleted after action a7, and thus, this object would be activated with a new ID if it was re-detected.

Consequently, the reward function in the temporary death is defined as:

(10) $$\begin{array}{c} R_{td}\left(s,a\right)=y\left(a\right)\left(\begin{array}{c} \max\\ 1\leq j\leq M^{t} \end{array}\left(W^{T}\tau \left(T_{t}^{i},D_{t}^{j}\right)+b\right)\right), \end{array}$$

where y(a) = +1 if action a = $a_{5}$, and y(a) = −1 if a = $a_{6}$. j indexes Q candidate detections for data association.

#### 3.3.5. Reinforcement Learning

The tracking drift problem is highlighted in onboard, multi-vehicle tracking tasks. A learned policy was performed to handle the tracking robustness. The binary classifier with enforcement learning was trained offline in public KITTI datasets and self-collected datasets where each sequence was marked with ground truth. In the training process, each MDP took unique action as indicated by the ground truth trajectory. The goal in this part was training an MDP policy that could be used to track all these targets. Reinforcement learning defined a set of actions a∈A that made achieving the maximum reward possible. This policy was updated only when the MDP made a mistake in data association.

To obtain a max-margin classifier for data association, the training function is used as follows:

(11) $$\begin{array}{c} \underset{w,b,\xi }{\min}\frac{1}{2}{\left\|W\right\|}^{2}+C\overset{Q}{\underset{k=1}{\sum }}\xi_{k} \end{array}$$

(12) $$\begin{array}{c} \mathrm{subject}\;\mathrm{to}\;y_{k}\left[W^{T}\tau \left(T_{t}^{i},D_{t}^{j}\right)+b\right]\geq 1-\xi_{k}\;,\xi_{k}\geq 0,k=1,2,\ldots ,Q, \end{array}$$

where $\xi_{k},k$ are the slack variables, and *C* is a regularization parameter. The policy was kept iterated when the classifier was updated until all the visible and correct targets were successfully tracked.

## 4. Experiments

**Figure 4:**
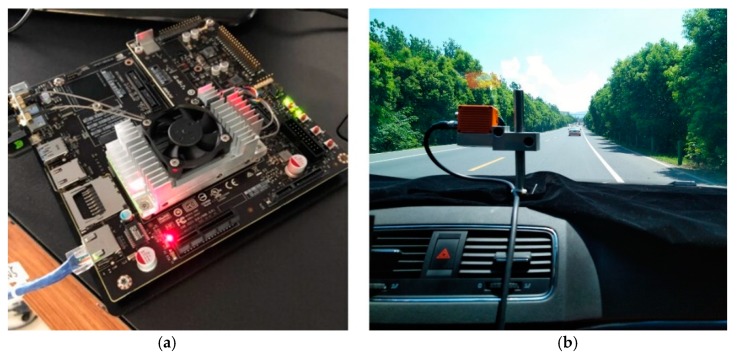
(**a**) NVIDIA Jetson TX2 with 256 GPU cores; (**b**) Comprehensive tests are validated in the moving vehicle in different scenes (e.g., highway).

In this section, dataset and evaluation metrics are presented in the first part. The comprehensive experiments were conducted in three stages. First, the comparison of different components was evaluated in three typical scenes on a self-collected dataset. Second, the motion and appearance models were disabled sequentially to evaluate the contribution of each component. Finally, the proposed method was compared with five state-of-the-art methods on KITTI datasets to assess the contribution of the work in terms of six evaluation metrics. As shown in Figure 4, comprehensive tests and analyses were performed on NVIDIA Jetson TX2 with an on-board camera.

### 4.1. Dataset and Evaluation Metrics

Datasets.

To evaluate the performance of the proposed multi-vehicle tracking method, extensive experiments were conducted on the KITTI Vision Benchmark Suite dataset [38], which is the widely used benchmark for multiple vehicle tracking. The training dataset consisted of 21 sequences with 8008 frames, and the testing dataset consisted of 29 sequences with 11,095 frames. Despite the dataset having labeled eight different classes, only the class “car” was considered in our work. Especially, the KITTI dataset provided object detection as well as tracking results in a full-face perspective based on its comprehensive annotations. It was crucial to the research of tracking by detection with a frontal, onboard monotonous camera. In the self-collected datasets, 50 annotated sequences of three typical traffic scenes in various light conditions were acquired from a moving camera with 480 × 640 pixels. All sequences had a varying number of objects and lengths with unique motion scenarios. The differences of size and orientation, occlusion pattern, and illumination were considered in our datasets.

Evaluation metrics.

For quantitative evaluation, the average precision (AP) was first taken into account to evaluate detection performance. A widely accepted protocol, CLEAR MOT metrics [39], were adopted, which included multiple-object tracking precision (MOTP) and multiple-object tracking accuracy (MOTA). The MOTP measured the ability of the tracker to estimate precise object positions. Furthermore, fragmentation (FRAG), ID switches (IDS), mostly-tracked (MT), and mostly-lost (ML) were also indispensable in valuing the performance in MOT. ID switch happened when a ground-truth trajectory was matched with another wrong identity. The MT and ML represented the percentage of the ground truth trajectories covered by the tracker output for more than 80% in length or less than 20% in length, respectively. Identification F1 score (IDF1) was the ratio of correctly identified detections over the average number of ground-truth and computed detections, which evaluated identification precision.

### 4.2. Performance Evaluation

The combined multi-vehicle tracking frameworks were evaluated on the self-collected dataset, which contained different motion patterns on campuses, urban roads, and highways. The previous algorithms “SSD” [40] and “YOLOv3” [41] performed well in object detection domains. By switching partial components, Table 2 shows the performance of detection and tracking in three typical traffic scenes. The bold results present relatively better performance. 

The evaluation results note that better detection results led to better scores in tracking. In moving scenes, the size of the target vehicle varied while the distance changed. YOLO was relatively sensitive to the changing scale objects, and the generalization ability of objects with large-scale changes was poor. Detection probes trained in augmented vehicle dataset significantly improved the detection performance (measured as AP) under diverse scenes. The customized detector combined with the proposed tracking scheme could stay competitive in different environments.

In a campus environment, the tracking scenario was relatively simple, where most of the target vehicles were parked on the roadside. But on the urban road, inter-object occlusion and truncation frequently occurred due to cluttered traffic scenes. Facing traffic signals and lane marks, the motion of each vehicle became relatively complicated. In the urban traffic intersection, vehicles show different shapes in our view, The traffic flow became smoother on the highway, in which vehicles kept moving in the same direction with typical highway situations, like cruising, overtaking, following, etc. They were free from other distractions, e.g., pedestrians or bicycles.

The trade-off between accuracy and speed was quite tough in detection and tracking tasks. The offline, pre-trained detector on the portable NVIDIA Jetson TX2 with 256 GPU cores could achieve real-time performance while maintaining competitive tracking accuracy. As the computation speed depended on the number of targets in the video sequence, tests were applied in three typical traffic scenes and returned about 25 frames per second (FPS).

**Figure 5:**
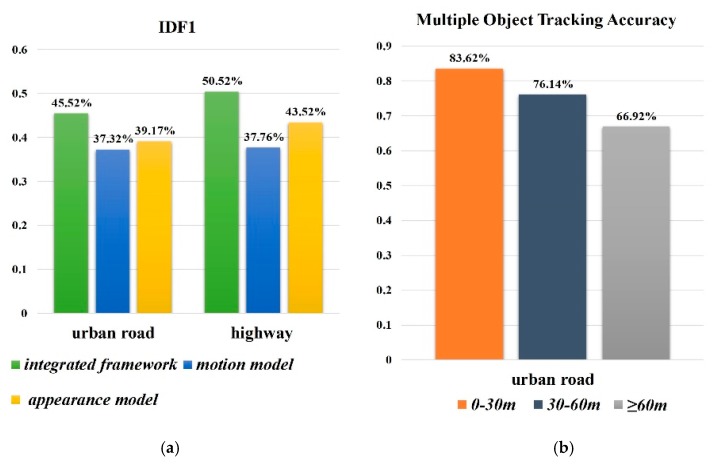
Comprehensive analyses of the proposed framework. (**a**) The contribution of each components in two typical scenes respectively; (**b**) The tracking accuracy in different distance and the threshold selection depends on the image size.

Inspired by the deep-sort method [27], only appearance information was used in the association cost term during the experiments when there was substantial camera motion. The motion model describes the movement of the object while the appearance model focused on the similarities of the surface features. In order to demonstrate the effectiveness of each component, the contribution of each model was investigated under two typical situations. Figure 5a illustrates the tracking performance under different situations in terms of IDF1 and MOTA. IDF1 is a major tracking metric that measures how often objects are correctly identified by the same tracking identity. As expected, significant performance drops happened when the single feature model was taken into account.

More specifically, tracking on the urban roads performs worse than on the highway because of the volume of road traffic facilities and inter-object occlusions. Appearance cues became less discriminative in over-crowed tracking backgrounds. One single cue was not reliable to capture the correlation of pairwise targets. The motion model only figured the relative location change, but it still had a gap in handling false positives near the target. Appearance constraints could significantly reduce this ambiguity. On the other hand, no motion model may contribute to the mishandling of target vehicles sharing the same characteristics. These limitations indicate that only considering both of the factors is sufficient to guarantee the robustness of MOT in dynamic and complex traffic scenes.

In terms of using the track method in the domains of intelligent vehicles to increase safety, the distance between the ego-vehicle and other objects is worth taking into account. Three distance thresholds were observed and analyzed in an urban road environment. The threshold selection depended on the image size in this test phase. As shown in the right histogram of Figure 5b, the multiple object tracking accuracy (MOTA) performed better when the targets were closer, in which they were highly threatened.

The proposed method was evaluated on the KITTI Tracking benchmark and only the “car” class was considered. A quantitative comparison between our method and other state-of-the-art tracking systems [42,43,44,45,46] is given in Table 3. Here, ↑ represents that higher scores indicate better results and ↓ notes lower are better. The bold results present relatively better performance.

The proposed method showed strong competition with other multi-object trackers. In particular, the number of “mostly tracked” increased by at least 8.89% while the FRAG, IDS, and other evaluated metrics were still robust.

**Figure 6:**
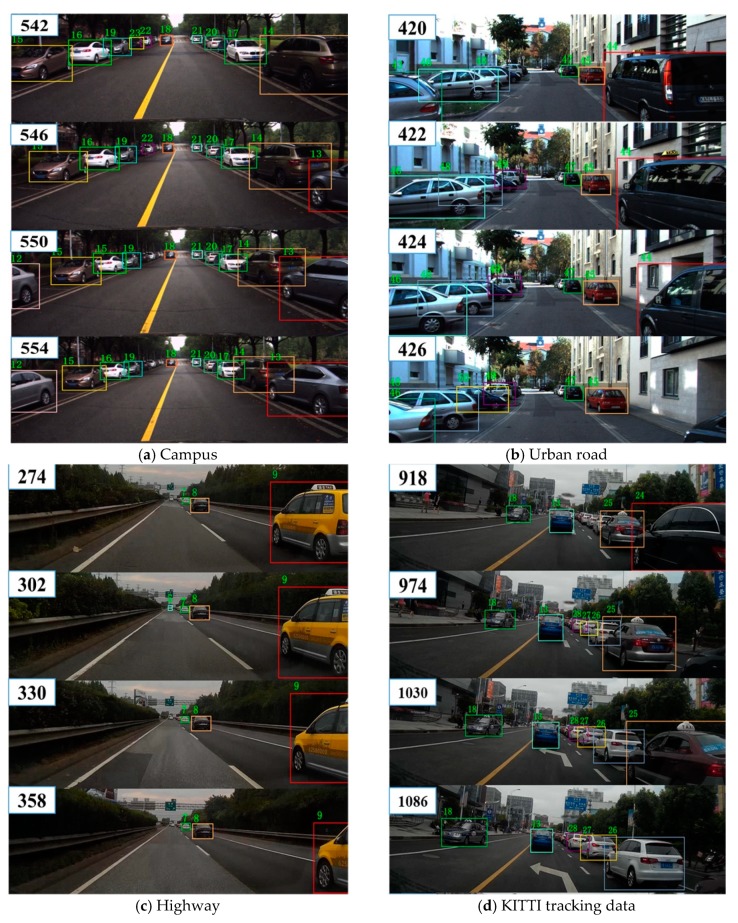
Exemplary output under four typical traffic scenes.

The high-precision detections can potentially reduce false positives and improve the tracking accuracy (measured as MOTA). The significant score of MT implied that this method could generate a more integrated trajectory. The result of identity switches was 11, which was really close to the best result of 7 the SSP algorithm. The ability to maintain target identity denoted that the tracking scheme could initialize and terminate targets effectively and keep robust trajectories, which was enhanced by the proper policy with reinforcement learning in MDP. The competitive comparison results verified the effectiveness of the multi-vehicle tracking method. The exemplary tracking results on campuses, urban roads, highways, and the KITTI dataset are shown in Figure 6.

## 5. Conclusions

In this paper, a novel method was customized to realize robust tracking of multi-vehicles with an onboard monocular camera in dynamic environments. Based on the tracking-by-detection framework, the detection probes were utilized to detect vehicles in real-time. A multi-feature model was designed to generate the matching matrix. The central-surround two-channel SPP (CSTCSPP) network generated discriminative similarity of appearance, while the motion model was used to account for the relative movements. Based on corresponding cues, the Hungarian algorithm helped to generate best matches in the data association process. Furthermore, to alleviate tracking drift, MDPs with reinforcement learning were implemented to transfer the state at each time step. The comparative experiments were conducted in different scenes to evaluate quality. The comprehensive performance analyses showed that our method was effective for real-time, long-term tracking and achieved an efficient improvement in robustness. In the future, we plan on expanding this application by adding more direction perspectives under different light conditions to employ in various scenes. 3D object detection, as well as related applications, will be considered in the next step, and the additional 3D object labels will be added to further improve the tracking performance. In addition, the system is planned to employ other specific kinds of objects, e.g., faces, pedestrians, and animals.

## Figures and Tables

**Table 1:** Details of each branch network.

Layer	Type	Kernel Size	Stride
**Input**	Raw data		
**Conv1**	Convolution	7 × 7	2
**Pool1**	Max pooling	3 × 3	2
**Conv2**	Convolution	5 × 5	2
**Pool2**	Max pooling	3 × 3	2
**Conv3**	Convolution	3 × 3	1
**Output**	FC		

**Table 2:** Comparative results under different traffic scenes.

Detector	Evaluation of Detection (AP)			Tracker	Evaluation of Tracking (MOTA)		
	Campus	Urban	Highway		Campus	Urban	Highway
SSD	65.25%	60.16%	68.84%	Proposed	70.64%	72.62%	74.32%
YOLOv3	63.55%	62.99%	70.19%	Proposed	74.65%	**77.22%**	77.98%
Detection probes	**68.84%**	**63.66%**	**72.03%**	Proposed	**75.29%**	76.06%	**78.14%**

**Table 3:** Comparison of our proposed methods with five state-of-the-art methods on KITTI.

Method	MOTA ↑	MOTP ↑	FRAG ↓	IDS ↓	MT ↑	ML ↓
Proposed	76.53%	81.19%	**349**	11	**82.12%**	9.92%
SSP [39]	57.85%	77.65%	704	**7**	29.38%	24.31%
RMOT [40]	65.83%	75.42%	727	209	40.15%	9.69%
MDP [41]	69.35%	82.10%	387	130	52.15%	13.38%
ExtraCK [42]	79.99%	82.46%	938	342	62.15%	5.54%
MOTBeyondPixels [43]	**84.24%**	**85.73%**	944	468	73.23%	**2.77%**
